# A Comprehensive Analysis of Wait Times in Ambulatory Obstetrics and Gynecology Clinic at a Tertiary Care Hospital in Pakistan

**DOI:** 10.7759/cureus.69442

**Published:** 2024-09-15

**Authors:** Safa Kamran, Salman Imami, Saima Jabeen, Abubakar Farooq, Shiza Zafar, Fozia U Qureshi

**Affiliations:** 1 Healthcare Management and Innovation, Lahore University of Management Sciences, Lahore, PAK; 2 Obstetrics and Gynecology, Shalamar Hospital, Lahore, PAK; 3 Obstetrics and Gynecology, Shalamar Medical and Dental College, Lahore, PAK; 4 Medicine, Fatima Memorial Hospital, Lahore, PAK

**Keywords:** ambulatory clinic, obstetric and gynecology, outpatient department, patient pathways, patient satisfaction, wait times

## Abstract

Objective

Wait times are an important determinant of patient satisfaction measured objectively and associated significantly with it. It is a measure of time taken from the arrival of patients to the registration desk of a facility to the time they access service. This study focuses on current patient pathways at the Obstetrics and Gynecology Outpatient (OBGYN) Ambulatory Clinic at Shalamar Hospital and assesses wait times by stratifying it at each step of the pathway.

Methodology

Data on consultation times was obtained from 105 patient processes over three months. We had in-depth discussions with the doctors, nurses, and paramedical staff to better comprehend the medical conditions, the terminologies, the structural hierarchy, and the functions of the department. Based on this the patient journey through the clinic was further dissected into operational steps including the check-in process, patient vitals screening, patient history, physical examination, medical hierarchy, referral for further testing or procedures, and lastly prescription generation.

Results

The average wait time for obstetrics and gynecology patients is 45.18±39.37 minutes. The average time the patients waited for the check-in process was 1.73±1.80 minutes, for the vitals screening was 3.08±10.07 minutes, for patient history and examination was 19.90±22.71 minutes, and for consultation within the medical hierarchy (SR and Consultants) patients waited 1.31±5.71 minutes. For those patients who had no other tests or procedures recommended, the prescription generation required a further 4.10±3.14 minutes. While those who had tests or procedures recommended waited 5.75±28.27 minutes for voucher generation for the specific test or procedure and 9.31±11.35 minutes for the test or procedure itself.

Conclusion

The obstetrics and gynecology clinic at Shalamar Hospital has average wait times comparable to others in the region of approximately 45 minutes. The longest wait during the visit is before patient history and examination approximately 20 minutes. To improve wait times, the clinic should implement staggered appointments, use a token system, manage queues better, follow clinic hours, and improve coordination between doctors. This will allow for improved patient experiences, faster patient turnovers, and increased patient inflow.

## Introduction

Patient satisfaction has been explained in Donabedian’s theory of healthcare quality as a positive evaluation of aspects of healthcare quality [1]. Wait time is one of the determinants of patient satisfaction and is the only determinant significantly associated with satisfaction [1]. There is no universal definition of wait time, and it is interchangeably used for the time taken to access care or the time taken to access the service [2]. However, an agreed-upon measurement of wait time begins when the patient arrives at the registration desk of a facility at the time they access the service [3].

As the population's access to healthcare grows, the demand for services intensifies, particularly in specialty clinics, which often manifests as longer wait times [4]. The average wait time for a patient in the United States (US) for an obstetrics clinic is 26 to 27 minutes, while in another study it was shown to be 32 minutes [5,6]. Similarly, average wait times were 30 and 45 minutes in English and Belgian studies [7,8]. In Malaysia, however, the wait times were longer, averaging 1 to 2 hours [4].

Studies show that when patients arrive at the clinic, they expect to wait, but the meantime they expect to wait is 30 minutes. As the time exceeds this threshold, they start becoming dissatisfied with the services of the clinic [4,9]. Although wait times are a key factor in evaluating patient satisfaction, other determinants include the quality of the waiting area, availability of entertainment or educational materials, the attitude and behavior of healthcare providers, and the duration of contact with providers during consultations [4,10]. There is an inverse relationship between wait times and patient satisfaction, and it is seen that patients who are not satisfied with the services are less likely to comply with treatment [9].

In our Pakistani setups, few single-centered studies have been done to assess the wait times in the obstetric and antenatal clinics, which have shown the wait times to exceed one hour [11,12]. The study done in Peshawar shows that more than 50% of patients had to wait more than one hour, and amongst them, 6% waited for more than four hours before they were examined by healthcare providers [11]. Similarly, the average wait time in a comparative study in Kharian before implementing a triage intervention was 194 minutes [12].

While maternal mortality remains high in the urban settings of Pakistan at 14% and still a large population of 43% prefers home birth, factors need to be identified that prevent access to healthcare [13]. Improving wait times in the obstetrics and gynecology clinics may improve attendance and make them more acceptable and efficient [9]. In the face of contagious disease, shortened wait times can prevent crowding and patient contact time, reducing the probability of disease spread [9].

Identification of points of delay in the patient pathway is the primary step in addressing the challenge of increased wait times. The study aims to provide the current patient pathways at the Obstetrics and Gynecology Outpatient (OBGYN) Ambulatory Clinic at Shalamar Hospital, Lahore, Pakistan, assess wait times by stratifying them at each step of the pathway, and assess the factors contributing to increased wait times.

## Materials and methods

Before starting the project the layout of the clinic was studied. We had in-depth discussions with the doctors, nurses, and paramedical staff to better comprehend the medical conditions, the terminologies, the structural hierarchy, and the functions of the department. The IRB approval was exempt for this study as this was a purely observational study with no interaction with the patients.

The data was collected between April and May 2023. One hundred patient journeys were planned to be closely monitored during the study period. The participants were randomly chosen at various times throughout the day during clinic time, and their journey was time-stamped. One researcher followed every patient from start to end noting the times at which the patient faced delays. A stopwatch was utilized to record the time. The primary data was collected by hand and then transcribed into Microsoft Excel files at the end of each workday for analysis.

**Figure 1:**
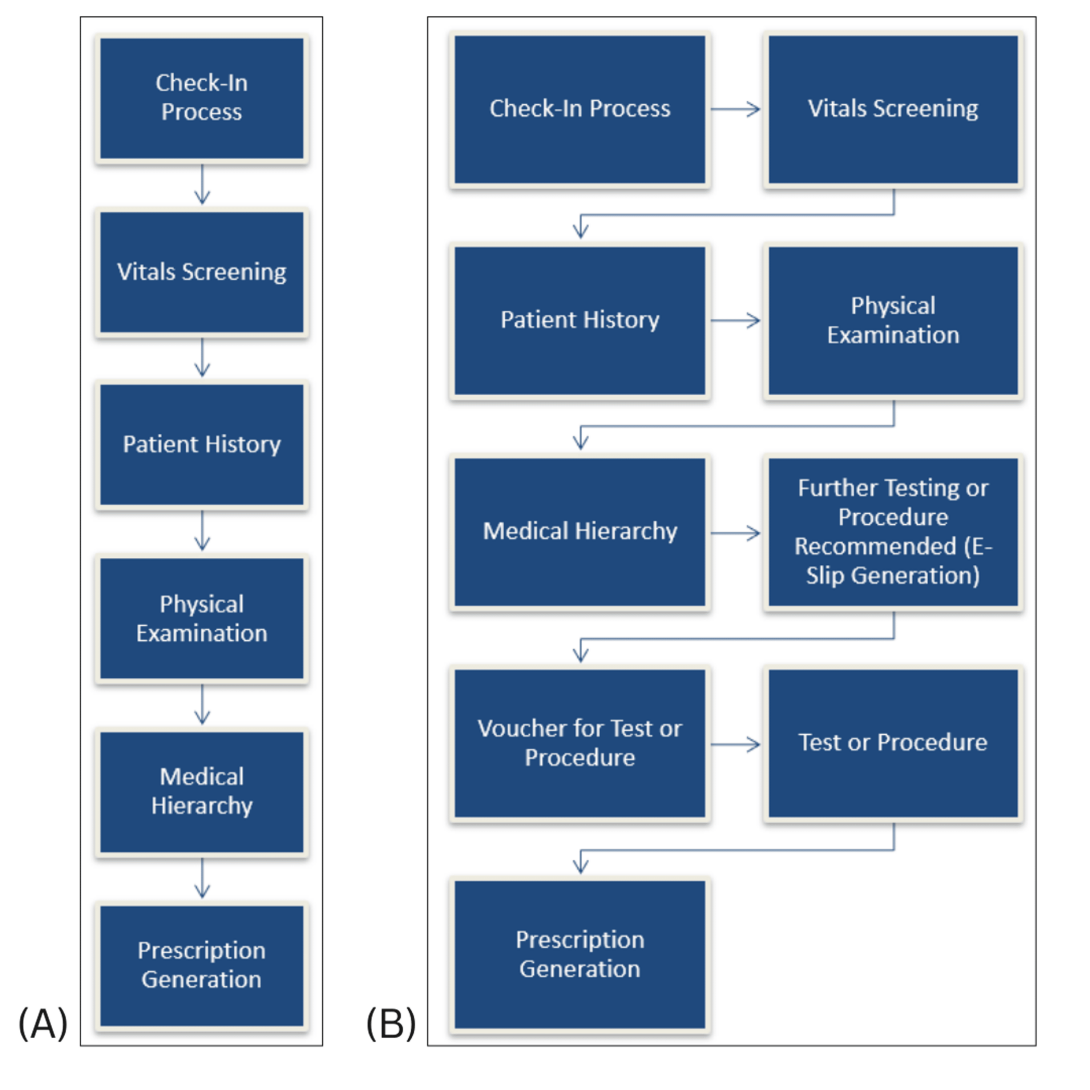
Patient journey through the ambulatory obstetrics and gynecology clinic (A) describes the first pathway that is taken by patients who visit the clinic and do not require additional procedures or testing till prescription generation. (B) describes the second pathway that is taken by patients who visit the clinic and require additional procedures or testing till prescription generation.

The data was populated into the following headings: date and time of patient entry, the diagnosis of the patients, time taken for each step throughout the journey in the clinic, patient waiting times at each step of the journey, and the designation of the clinical and support staff tending the patient at each stage. The entire patient journey through the clinic was further dissected into operational steps including the check-in process, patient vitals screening, patient history, physical examination, medical hierarchy, referral for further testing or procedures, and lastly prescription generation as seen in Figures 1A, 1B. The first pathway is taken by patients who do not require further testing and/or procedures and the second pathway is taken by patients who require these additional tests and/or procedures.

Check-in process

The first step of the patient journey involves checking in at the reception desk. Here, the patient pays the consultation fee. After payment, they receive a token slip, and depending on how busy the clinic is, patients may be directed straight to the vitals screening unit or asked to wait in the designated waiting area.

Vitals screening

The next step is vitals screening. In a dedicated room, two nurse assistants will measure the patient’s weight, height, blood pressure, heart rate, and temperature. For pregnant patients or new obstetrics patients, this information is recorded on a blue card. Existing obstetrics patients bring their blue cards for updates. Gynecology patients have their vitals noted on the token slip they received at reception.

Patient history and physical examination

Once vitals are complete, patients may be asked to wait for an initial screening by a junior doctor (house officer (HO)) or resident (postgraduate resident (PGR)). During this initial consultation, the doctor will take a complete medical history. Following the initial consultation, patients are taken for a physical examination in a separate examination room.

Medical hierarchy

It's important to note the medical hierarchy within the clinic. HOs are junior doctors who may consult with senior PGRs for complex cases. If further expertise is needed, the PGRs may consult with a senior registrar (SR) and ultimately, a consultant. This ensures that the patient receives the most appropriate care for their situation.

Tests and procedures

If any tests or procedures, like an ultrasound, PAP smear, etc., are necessary, a transcriptionist will print an e-slip for the patient. If no tests are needed, patients receive an e-slip with their prescription and are free to leave the clinic. However, if an e-slip for a test or procedure is issued, patients need to visit reception again to receive a voucher for payment before the test or procedure itself is conducted.

Following the data collection that was carried out over two months, data was analyzed using Microsoft Excel. Descriptive statistics were utilized to describe the continuous data of wait times.

Inclusion criteria

Data of individuals aged 18 years and above attending the Obstetrics and Gynecology Clinic between the timeframe of April to May 2023. Both newly diagnosed patients (those receiving their diagnosis or having their first confirmed visit) and established patients (those on follow-up appointments) were encompassed in the study.

Exclusion criteria

Those individuals whose data was missing or incomplete were excluded.

## Results

A hundred and five patient journeys were analyzed between April and May 2023. There was no missing data for any of the individuals. The average number of patients per month that seek care at the clinic is around 4000, with the maximum number of patients during the study period per day amounting to 196 per day. The clinic was functional six days a week from 8 am to 3 pm, with an exception on Friday when the timings were 8 am to 1 pm.

**Figure 2:**
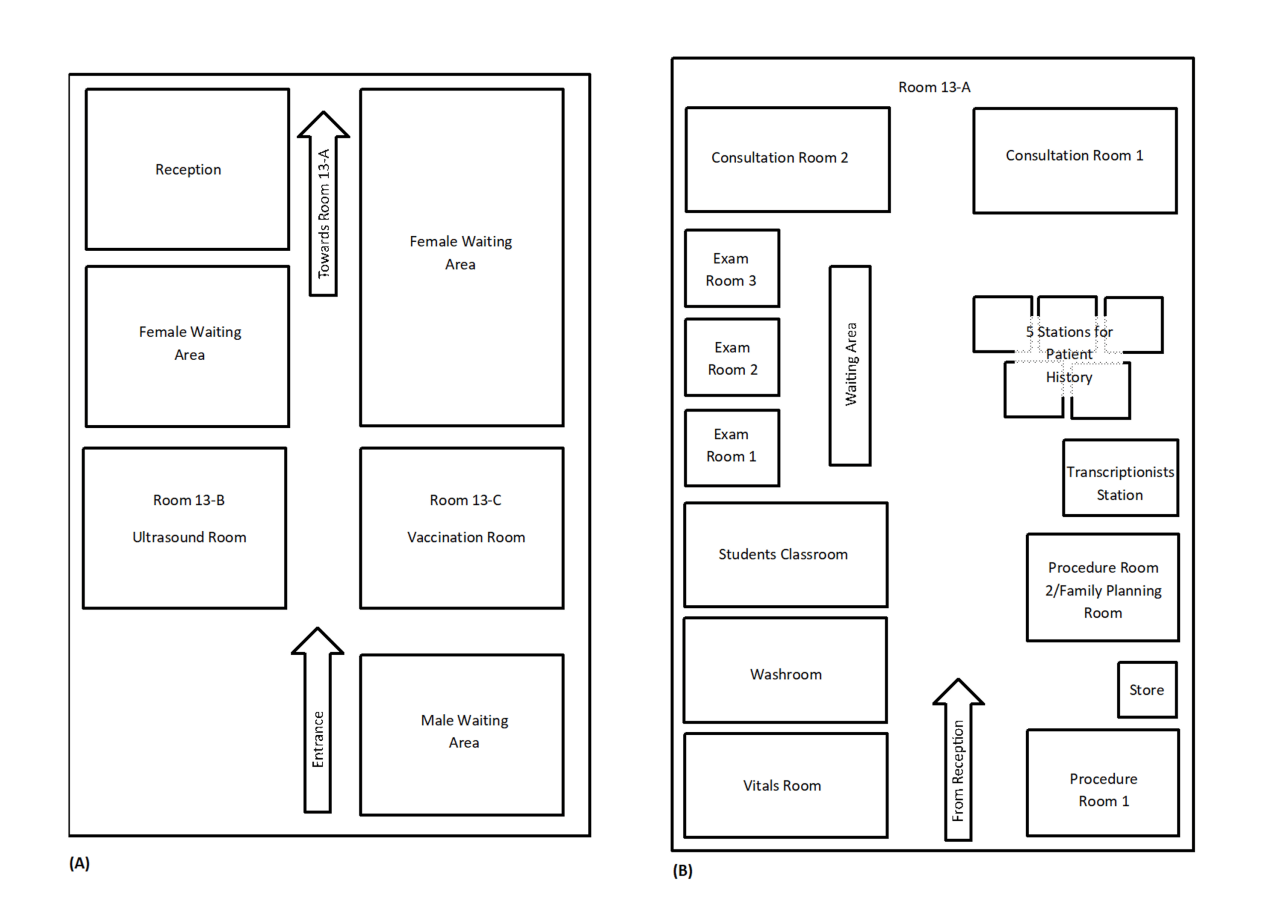
Layout of the ambulatory obstetrics and gynecology outpatient clinic (A) shows the entrance, the waiting areas, the reception, and the ultrasound (room 13-B) and vaccination rooms (room 13-C). (B) is a continuation of the patient's journey in room 13-A, which is further subdivided into multiple areas.

The layout of the clinic is given below in Figure 2. The ambulatory outpatient clinic comprises three rooms: a 13-A consultation unit, a 13-B ultrasound room, and a 13-C vaccination room. As the patient enters they move to the reception for check-in. If the patient is accompanied by a male attendant, the attendant is asked to wait in the male waiting area (Figure 2A). Depending on the patient load the patient is either asked to wait after check-in or they move for vitals screening. Patients then move to stations for patient history, where a detailed consultation with junior HO is held, which is supervised by the PGRs (Figure 2B). Patients are then taken into one of the three examination rooms for a thorough physical examination. According to the complexity of the cases, the SRs or consultants are taken on board, where the consultations are then held in the consultation rooms after the preliminary steps are completed. Last, if an ultrasound or a procedure is advised the patients return to the reception to generate a voucher, while if the patients are not advised of any tests or procedures they are sent to the transcriptionist who generates the electronic prescription.

The consultation unit 13-A also has a dedicated student classroom for medical students, a store, and a washroom for patients where urine may be collected for testing. There is also a patient waiting area for those awaiting history, examination, or consultation.

The average number of patients per day was 114±48. While there was a greater than average load on Mondays (128±31), Thursdays (157±38 patients), and Saturdays (158±30 patients) in patient volume. Of the patients who were observed 67% had an obstetric diagnosis and 33% had a gynecological issue. The most common reason for patients to visit due to an obstetric cause was antenatal care follow-up while the most common reason for visit due to a gynecological cause was polycystic ovarian syndrome (PCOS) (Table 1).

**Table 1:** Obstetrics and gynecology patients diagnostic codes IUGR: intrauterine growth retardation; IUCD: intrauterine contraceptive device; PCOS: polycystic ovarian syndrome; HVS: high vaginal swab

Obstetrics	
Diagnostic code	Number of patients
Gestational diabetes mellitus	7
Antenatal care/initial pregnancy	6
Antenatal care/follow up	20
Antenatal care/tetanus vaccination	3
Antenatal care/steroid injection	5
Blood sugar random	7
Breastfeeding complications	1
Doppler test for IUGR	7
Pregnancy-induced hypertension	6
Stitches removal	8
Total	70
Gynecology	
Diagnostic code	Number of patients
Dysmenorrhea	7
Irregular menstruation disorder	1
IUCD insertion	3
IUCD removal	3
Pap-smear + HVS	4
Pap-smear	1
PCOS	9
Urinary tract infection	5
Ultrasound genital tract	1
Routine gynecological checkup	1
Total	35

The average wait time for obstetrics and gynecology patients is 45.11±39.37 minutes. The wait times per step are given below in Table 2. The average time the patients waited for the check-in process was 1.73 minutes, for the vitals screening was 3.08 minutes, for patient history and examination was 19.90 minutes, and for consultation within the medical hierarchy (SR and Consultants) patients waited 1.31 minutes. For those patients who had no other tests or procedures recommended, the prescription generation required a further 4.10 minutes. While those who had tests or procedures recommended waited 5.75 minutes for voucher generation for the specific test or procedure and 9.31 minutes for the test or procedure itself.

**Table 2:** Wait times for each operational step stratified by type of patient

Operational step	Overall patient wait times (mean±SD)	Obstetrics patients wait times (mean±SD)	Gynecology patients wait times (mean±SD)
Check-in process	1.73±1.80	1.86±2.02	1.55±1.45
Vitals screening	3.08±10.07	4.72±12.57	0.79±3.94
Patients history and examinations	19.90±22.71	19.51±19.62	20.45±26.64
Medical hierarchy	1.31±5.71	2.16±7.36	0.136±0.76
Voucher for test or procedure	5.75±28.27	8.09±36.98	2.5±2.12
Test or procedure	9.31±11.35	8.95±10.32	9.18±12.74
Prescription generation	4.10±3.14	4.82±3.45	3.25±2.42

## Discussion

Our study comprehensively analyzes each step in the clinical consultation pathway for both obstetrics and gynecology patients. Combined average wait times for both of these patients provide a valuable statistic as these patients are seen in a queue regardless of condition (obstetrics or gynecology). While the developed world has seen advancements in improving patient satisfaction, wait times contribute immensely to patient dissatisfaction.

**Figure 3:**
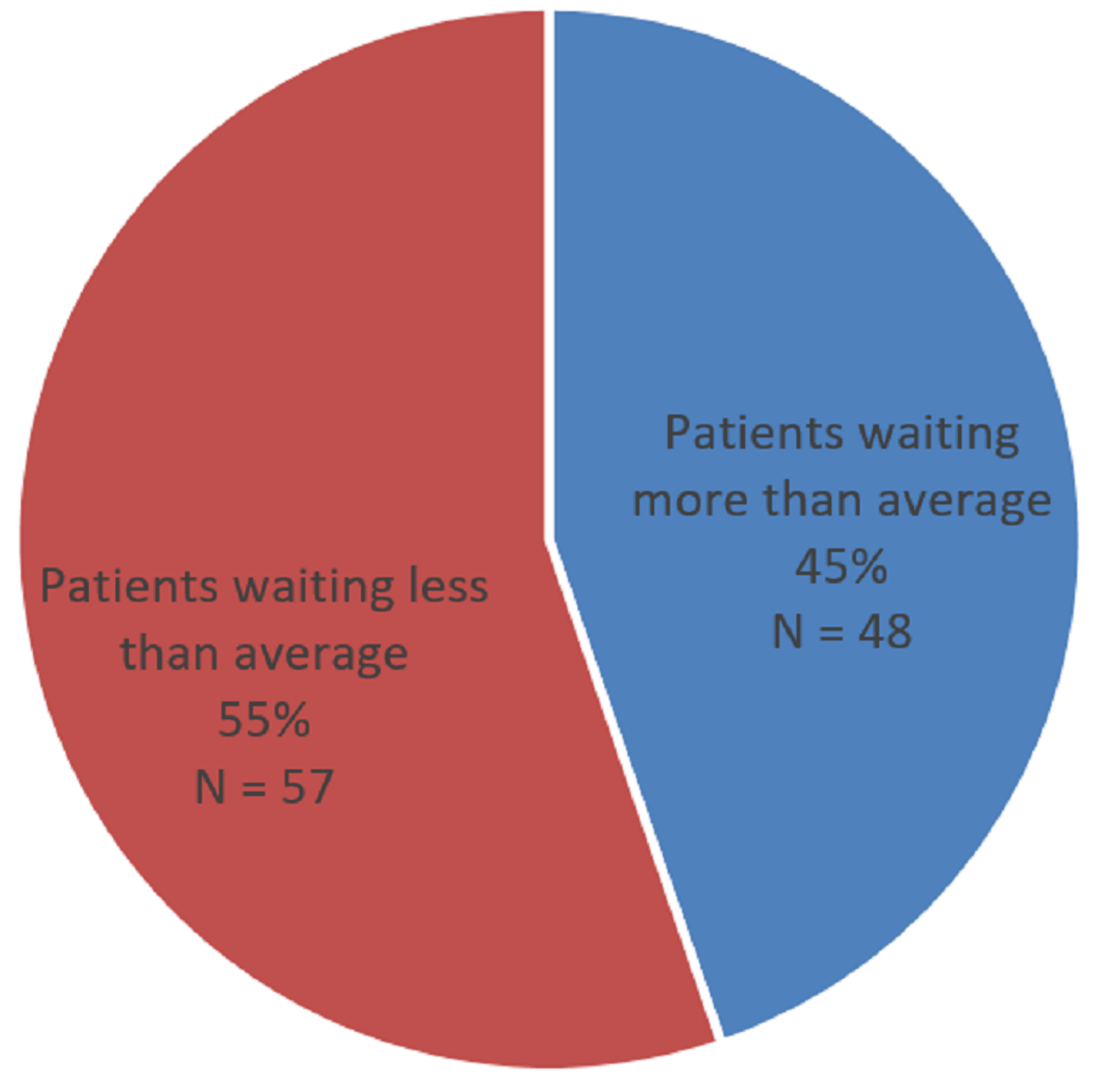
Percentage of patients waiting for more than the average wait time

In the United States and Britain, it was seen that the average time the patient had to wait upon arrival at a clinic before being seen by a physician was between 26 and 30 minutes [5-7]. In our study, this wait time has come out approximately twice (45 minutes) as much, showing room for improvement. However, this is still shorter than many other developing countries and other setups within Pakistan where wait times range from one to four hours [4,11,12]. Most of the patients were seen within the average calculated wait time; 45% were seen to take more as shown in Figure 3.

In our literature review, we found no studies that broke down the steps of the ambulatory outpatient consultation in a comprehensive manner as ours except for another study done at the same hospital measuring the consultation times [14]. The segment of the consultation where the patients had to wait the most was before "patients history and examinations," for approximately 20 minutes. This time adds to the overall wait time and was highlighted as the bottleneck, which should be addressed to improve patients experience in the clinic. While our study does not directly measure patient satisfaction, literature has shown us that waiting for more than 30 minutes increases patients’ dissatisfaction [4].

Multiple factors contributed to the bottleneck mentioned above. Doctors' advice on logistical matters, such as scheduling and queuing, coupled with inefficient token generation and queue management, created delays. Uncommunicated consultant schedules, varying break times, and overlapping non-clinical duties among consultants and senior staff further exacerbated the issue. Additionally, a lack of streamlined communication between the HO and PGR hindered the timely escalation of care, leading to longer wait times.

The issues that were identified were primarily logistical or managerial but cultural element played its role as well. While the clinic gave out appointments and started accepting patients from 8 am, most of the patient load was walk-ins. Additionally, these patients chose a time of their convenience between 11 am and 12 pm, leading to an extremely busy clinic and very high wait times, with some outliers coming around 285 minutes (five hours).

Currently, no universally agreed-upon metric for wait times is followed. Moreover, the patients' perceived wait time may be influenced by many factors which may be personal or based on socio-demographic features [15].

Recommendations

Staggered Appointment

While we acknowledge the cultural shift required in the population for patients to schedule appointments, incentivized appointments can be given during those hours when the clinic is less busy, early morning and late afternoon [9].

Proper Check-In Through Token System and Queue Management

While a system is in place for queueing and examining patients according to token number printed on the slip during check-in it is not followed. Ensuring adherence to tokens and queue management will lead to reduction in overall wait times and improved patient satisfaction. Furthermore, online systems of token generation have been utilized. These will reduce the number of persons waiting in the waiting area, as getting live updates of the current token number will allow the patients the freedom to move around and complete other tasks, reducing the perceived wait times [16].

Adherence to Timings and Accountability

The clinic begins from 8 am and ends at 3 pm. However, doctors often schedule non-clinical tasks within this time, leaving patients waiting to be seen adding to wait times. The doctors should adhere to the given times so patients can be seen as quickly and thoroughly as possible. Furthermore, break times for the doctors are not fixed. To improve patient confidence a fixed break should be given, where the clinic is not completely shut down but works at a smaller capacity allowing for patients to be seen and the wait times to be reduced. An administrative check should be done randomly to ensure that all doctors are at the assigned workplace.

House Officers and Postgraduate Resident Coordination

The HO often requires the PGR to review their case, and this leads to a delay if the PGR is busy managing their own patients. There is no fixed ratio such as one PGR to two HOs, and the HO can go to any PGR they like. This leads to overburdening of some PGRs while leaving other resources underutilized. A fixed HO and PGR unit should be created for a time so that there is mutual understanding between HO and PGR regarding each other’s responsibilities.

Strengths and limitations

This is one of the first studies conducted in Pakistan stratifying the consultation experience to investigate the delays and overall contribution to wait times in an obstetrics and gynecology setup. The strength of this study lies in its real-world data collection without any input from the researchers. The staff at the clinic including doctors, nurses, HOs, and PGRs, were all blinded. They were not aware of the patients that were being followed to minimize bias.

Since our study was observational and data was collected in a non-randomized manner, based on researchers' convenience, we cannot effectively rule out the element of selection bias. We conducted this study at a single ambulatory clinic in a not-for-profit hospital, which may affect the generalizability of our study since all patient populations were not equally studied.

A future study with a more robust methodology is planned to assess the utility of the recommendations that were given based on this study to improve the patient journey and flow within the clinic. It will also assess patient satisfaction in each step of the consultation process and will be carried out over an extended period. These future studies may help us evaluate patient perceptions of wait times as well.

## Conclusions

The wait times in the ambulatory obstetrics and gynecology clinic in Shalamar Hospital are comparable within the region, averaging 45 minutes. While the breakdown of the consultation process has revealed that the step of patient history and examination has the greatest individual wait time of 20 minutes. Ensuring adherence to the recommendations given, such as implementing staggered appointments, following a token system and proper queue management, adherence to clinic timings, and enhanced coordination between HOs and PGRs, will result in an improvement in wait times. This will further allow for improved patient experiences, faster patient turnovers, and increased patient inflow.
